# Investigation of enzymatic hydrolysis kinetics of soy protein isolate: laboratory and semi-industrial scale

**DOI:** 10.1186/s40643-022-00518-2

**Published:** 2022-04-04

**Authors:** Nikita Pozdnyakov, Sergey Shilov, Alexander Lukin, Maxim Bolshakov, Evgeny Sogorin

**Affiliations:** 1 Federal Research Center “Pushchino Scientific Center for Biological Research of the RAS”, Institute for Biological Instrumentation, Institutskaya Street, 142290 Pushchino, Russia; 2 Federal Research Center “Pushchino Scientific Center for Biological Research of the RAS”, Institute of Basic Biological Problems, Institutskaya Street, 142290 Pushchino, Russia

**Keywords:** Soy protein isolate, Protosubtilin, Alcalase, Subtilisin, Enzymatic hydrolysis, Semi-industrial scale

## Abstract

Using parameters of optimal conditions from laboratory experiments often results in the loss of significant time and resources when trying to scale up the process. In this study, the comparison of results of laboratory and semi-industrial experiments of enzymatic hydrolysis of soy protein isolate is considered. The kinetics of peptides accumulation was investigated by colorimetric method in both microtube (volume reaction is 0.7 ml, 7.14 mg/ml of substrate, incubation in solid state thermostat) and industrial homogenizer (volume reaction is 4,000 ml, 100 mg/ml of substrate, rotor–stator type mixer). The enzyme preparation Protosubtilin G3x (main component is subtilisin) was used as an analogue of the Alcalase preparation, which is already widely used in the food industry. It was found that the pH and the number of proteolytic units in the reaction mixture of both scales had slightly different results of the kinetics, while the temperature showed significantly one. The laboratory scale of the reaction had a wide range of optimal temperature (40–60 ^∘^C, 30 ^∘^C showed slowest rate of kinetics reaction), whereas the semi-industrial scale had 50 ^∘^C of optimal temperature (30, 40, 60 ^∘^C had the same kinetics). It also was found that maintaining the pH value of the reaction mixture was not mandatory. The obtained results indicate the need to refine the process conditions using semi-industrial experiments before attracting industrial-scale resources. In the case of selection of conditions for the hydrolysis of soy protein isolate in production, it is necessary first of all to take into account the reaction temperature as the most irreproducible parameter when scaling.

**Graphical Abstract:**

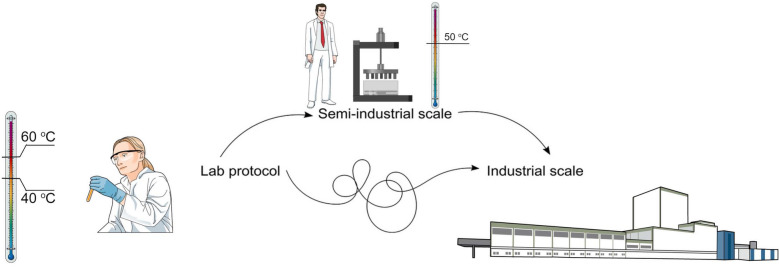

## Introduction

The consumption of animal food products is constantly increasing due to the growth of the world’s population. The expansion of animal husbandry leads to an increase in the load on natural systems accompanied by a number of economic and environmental problems (Godfray et al. 2018). The search for alternative sources of protein will allow us to satisfy the people’s need for protein without further livestock growth and related problems (Thavamani et al. 2020).

The production of plant protein as an alternative to meat protein requires less resources due to the lack of a stage of feeding agricultural animals, which are raised mainly for meat. In addition, the production of plant protein has a low cost compared to meat production (Willett et al. 2019). Also, the increasing popularity of vegetarian diet stimulates manufacturers to search for the new forms of food products obtained from plants, including plant proteins (Thavamani et al. 2020). The main sources of plant-based protein are cereals, legumes, oilseeds, roots and tubers, nuts. Various plant-based isolates and concentrates can be found on the market, such as wheat, pea, potato, corn, soy etc. (Kumar et al. 2021)

The work (Gorissen et al. 2018) presents a detailed comparative characterization of the amino acid profiles of commercially available plant-based and animal proteins. Despite the fact that plant proteins are limited in the content of some amino acids (mainly methionine, lysine and threonine), plant raw materials have a high potential to fill the protein deficiency in the human diet (Gorissen et al. 2018; van Vliet et al. 2015).

**Fig. 1:**
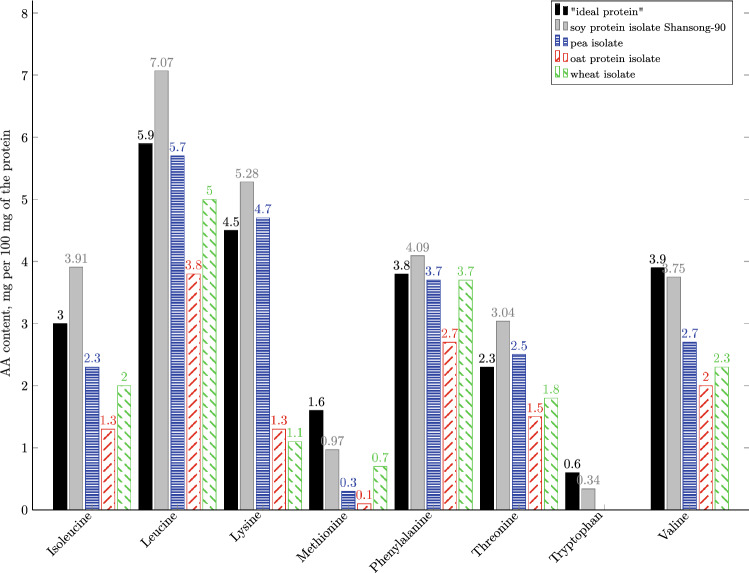
Amino acid profiles of commercially available plant-based proteins The graph was made using the following sources: “ideal protein” [7], soy protein isolate Shansong-90 (Muranova et al. 2018), pea isolate (Gorissen et al. 2018), oat protein isolate (Gorissen et al. 2018) and wheat isolate (Gorissen et al. 2018)

**Fig. 2:**
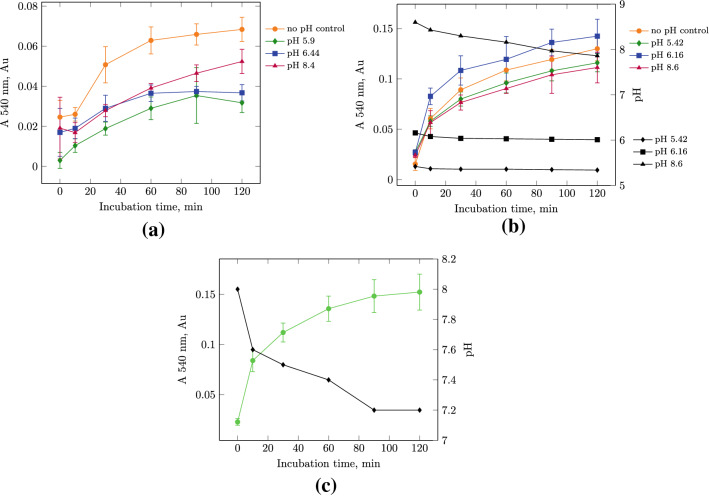
Kinetic dependence of the TCA-soluble peptides accumulation on the pH of the reaction mixture during the enzymatic hydrolysis of soy protein isolate (SPI) under the action of protosubtilin. **a** On a laboratory scale; **b** on a semi-industrial scale with buffer systems; **c** on a semi-industrial scale with water (11.9 units per 1 g of SPI, 40 ^∘^C). Changes in the pH of the reaction mixture during the reaction in buffer system are shown by the black curves

**Fig. 3:**
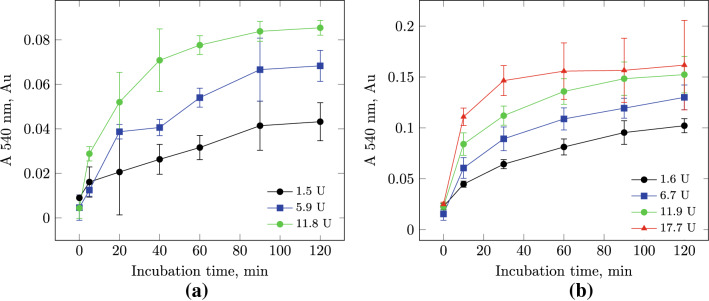
Kinetic dependence of the accumulation of TCA-soluble peptides during the hydrolysis of soy protein isolate (SPI) on the number of protosubtilin activity units (PS) (40 ^∘^C, in deionized water). **a** On a laboratory scale; **b** on a semi-industrial scale

Soybeans contain 35–50% of protein (by dry weight), which leads to their widespread use for food and forage around the world. Based on data on the daily intake of amino acids, the Food and Agriculture Organization of the United Nations (FAO) and the World Health Organization (WHO) proposed the “ideal protein” [7] based on human muscle protein as reference. Analysis of the distribution of essential amino acids in soy and other plant proteins showed that soy protein is similar to the “ideal protein”, only methionine is slightly scarce (Fig. 1). The high content of lysine, as well as the presence of flavonoids, phytosterols, polyunsaturated fatty acids allows to use soy and by-products of its processing in the production of special dietary foodstuffs and preventive nutrition (Clemente 2000; Messina et al. 2017).

Soy raw materials are characterized by high protein content and low fat content, which is an advantage compared to food products of animal origin. However, the use of soy protein as a food product has a number of issues. One of them is the presence of inhibitors of the proteolytic enzymes pepsin, trypsin, chymotrypsin, human gastrointestinal lipase (Mosolov 1982; Clemente et al. 2019; Petibskaya 1999; Kononova et al. 2016), which leads to a decrease in protein digestibility. In addition, plant raw materials contain a number of other substances of a non-protein nature that have a negative impact on the human body. In particular, saponins lead to hemolytic processes of red blood cells, genistin blocks calcium metabolism, oligosaccharides (stachyose, raffinose) cause flatulence, and lectins inhibit the action of maltase and aminopeptidase (Kononova et al. 2016; Vozyan et al. 2013). To increase the digestibility of plant protein, protein concentrates and isolates are prepared from raw materials. Also the activity of proteolytic enzyme inhibitors is reduced by exposure to high temperatures (DiPietro and Liener 1989; Birk 1961; Ellenrieder et al. 1980; Nordal and Fossum 1974; Muranova et al. 2019).

A promising direction for increasing the digestibility of vegetable raw materials is the cleavage of protein macromolecules to low-molecular peptides and free amino acids, i.e., the production of protein hydrolysates. Some studies were conducted earlier (Muranova et al. 2019; Zinchenko et al. 2018). In this works, the enzymatic hydrolysis of soy and rapeseed protein isolate with the enzyme preparation Protosubtilin G3x (PS) (Sibbiopharm, Russia) was considered. (PS is the culture fluid of *Bacillus subtilis* sprayed on carrier powder. The main component of PS is a serine protease of bacterial origin subtilisin). Vegetable raw materials (0.3%) were being hydrolyzed for 20 h at room temperature at an enzyme/substrate ratio of 1:20 and 1:100 (weight/volume).

Also, the prospect of using the enzyme preparation of red king crab hepatopancreas in the hydrolysis of soy protein isolate has been shown recently (Muranova et al. 2018). A high degree of hydrolysis was achieved under conditions of prolonged hydrolysis (18 h) with 0.3–0.5% of the substrate in the reaction mixture. The ratio of the enzyme to the substrate was from 1:5 to 1:100 by protein weight. It should be noted that such laboratory conditions of hydrolysis have little to do with the production conditions and do not provide a complete understanding of how the process will proceed when scaling.

The problem of transferring of laboratory protocols to a semi-industrial scale is well-known Reisman (1993). The amount of reagents, time of the reaction, charges for the electricity and work time are not usually considered in laboratory protocols but do matter in industry.

Many other protocols of soy protein hydrolysis using different enzyme preparations exist. Enzyme preparations that are used for this purpose include but not limited to proteases of plant origin (bromelain, papain, ficin) (US20070042104A1, EP0797927A1, US4563357, US20070014896A1, US20070042103A1, US20070042104A1), fungal proteases (Amano, Flavourzyme etc.) (US4632903, US7771762B2, US20020132287A1, US20050053705A1, US20120329985A1), proteases of animal origin (trypsin, chemotrypsin) (US5780439, RU2039460C1) and also bacterial proteases (Alcalase, Neutraze, Protex, Promatex) (US4100024, US4324805, US5716801, US7300680B2, US7332192B2, US20060134310A1, US20070269554A9, US20080096243A1, US20080182002A1, RU2011132127A, US4885178, US5520935). Also, there is a lot of patents with phrases like “any protease”, sometimes with some properties such as type and pH range, but without defining the concrete preparations (US4697004, US4107334, US6022702, US20060159826A1, US20070042103A1, US20070042104A1). Nevertheless, it is difficult to identify if these protocols has been tested on a semi-industrial or industrial scale or based on laboratory research only.

The most commonly used enzymes for hydrolysis of soy protein isolate on a semi-industrial scale were compared and it has been found that the highest hydrolysis level is observed when using the enzyme preparation Alcalase 2.4 L FG (Meinlschmidt et al. 2015). Alcalase is a commercial enzyme preparation obtained from *Bacillus licheniformis*. The main component is a serine protease of bacterial origin (subtilisin), firstly obtained from *Bacillus subtilis* Ottesen and Svendsen (1970).

As we know from the literature, there are no studies devoted to comparing of the hydrolysis conducted on a laboratory and semi-industrial and industrial scale using the same enzyme preparation for the same substrate. In this paper, for the first time, the efficiency of hydrolysis of soy protein isolate in the presence of a proteolytic enzyme preparation of microbiological origin is compared on a laboratory and semi-industrial scale using the Biuret reagent to detect TCA-soluble fraction of peptides. It is shown that on a laboratory scale the content of TCA-soluble peptides increases with the increase of proteolytic activity, the optimal temperature degree is 40–60 ^∘^C with deionized water instead of a buffer system. On a semi-industrial scale it is shown that there is no need to reach the PS activity more than 6.7 units/g of SPI, the optimal temperature is 50 ^∘^C and there is no significant difference between the results obtained in different buffer systems and deionized water. Also the distribution of molecular weights of peptides of SPI hydrolysates obtained in different conditions has been compared using size exclusion chromatography. The obtained data highlight the necessity of improvement of laboratory protocols for industrial conditions.

## Materials and methods

### Enzyme preparation

Commercial enzyme preparation Protosubtilin G3x (PS) (Sibbiopharm Ltd., Russia; 250 units proteolytic activity/g) is the culture fluid of *Bacillus subtilis* sprayed on powder carrier. That’s why filtration and purification steps are nessessary. PS was dissolved in deionized water to a concentration of 150 g per liter, thoroughly mixed for 15 min and filtered through a non-woven filter to remove insoluble large particles. The resulting solution was filtered using the AP-3-300 ultrafiltration module with hollow fibers (the pore size of the membrane was 300 kDa) (Scientific-Production Complex (SPC) Biotest, Russia). Furthermore, it was concentrated 6 times using an ultrafiltration module with hollow fibers AR-1-5 (the pore size of the membrane was 5 kDa) (SPC Biotest, Russia). The concentration of protein in the final enzyme preparation was 11.7 mg/ml. The preparation was stored at − 20 ^∘^C.

### Determination of proteolytic activity

The determination of proteolytic activity was carried out on the basis of the GOST 34430-2018 protocol with minor changes. 2% casein solution in a universal buffer (40 ml 0.1 M $CH_3 COOH$, 99.8%; 40 ml 0.1 M $H_3 BO_3$, 99%; and 40 ml 0.1 M $H_3PO_4$, 98%; brought to pH 8.0 with a 1 M NaOH solution) was used as a substrate. Control and experimental solutions of 100, 150, 200 and 400 fold diluted PS with a volume of 500 $\upmu$l so the obtained total protein concentrations of PS were 117, 78, 58.5 and 29.25 $\upmu$g/ml. The samples of PS were thermostated at 40 ^∘^C. 1 ml of 5% trichloroacetic acid (TCA) solution was added to the control solutions to inactivate PS before the substrate was introduced. To each of the PS samples, 500 $\upmu$l of a substrate thermostated at 40 ^∘^C was introduced and incubated for 10 min at 40 ^∘^C. The reaction in experimental samples was stopped by adding 1 ml of 5% trichloroacetic acid (TCA) solution to the experimental samples. After adding the TCA samples were incubated for 20 min and centrifuged at 12,300 g for 3 min for the sedimentation of TCA-insoluble fraction. The optical density in the TCA-soluble fraction was determined using the Lowry method Lowry et al. (1951). The enzyme activity was calculated by the formula:

1 $$\begin{aligned} \text {Units/ml} = \frac{(D\cdot 4)}{\text {TE}\cdot 10\cdot V}, \end{aligned}$$

D-optical density of the test solution; 4-the ratio of the volumes of the reaction mixture and the enzyme solution after the addition of TCA; TE-tyrosine equivalent; 10-substrate hydrolysis time, min; V-the volume of the enzyme preparation introduced into the reaction mixture.

The tyrosine equivalent was defined as the optical density of the sample with 0.1 mg/ml tyrosine determined using the Lowry method.

### Determination of the hydrolysis kinetics on a laboratory scale

Hydrolysis of 0.71% (7.14 mg/ml) soy protein isolate Shansong 90 (SPI) (Linyi Shansong Biological Products Co., Ltd., China; 90% protein) was carried out in microtubes in a solid-state thermostat. When studying the dependence of the hydrolysis rate on temperature, the temperature values of 30, 40, 50 and 60 ^∘^C were used; in other experiments the temperature of 40 ^∘^C was used.

500 $\upmu$l of 1% SPI solution and 200 $\upmu$l of PS solution were mixed in the microtubes.

In a series of experiments on the study of the dependence of hydrolysis rate on the activity of PS in the reaction mixture, the following activity values were used: 1.5, 5.9 and 11.8 units per 1 g of SPI. In the remaining experiments, the activity of PS in the reaction mixture was 6.7 units per 1 g of SPI.

To study the dependence of the hydrolysis kinetics on pH, a 1.25% solution of SPI was mixed with phosphate buffer (pH 6.00 and 5.29) and borate buffer (pH 8.62) with a concentration of 1 M in a ratio of 4:1. The chosen values of pH are the same as was on a semi-industrial scale after SPI addition to the buffers. pH of the reaction mixture hasn’t been measured during the reaction. The reaction was stopped after 10, 30, 60, 90 and 120 min by adding 400 $\upmu$l of 5% TCA. Deionized water was used instead of the enzyme preparation in the control samples. The contents of the tubes were mixed and incubated for 10 min. Then they were centrifuged for 3 min at 12,100*g*. 40 $\upmu$l of the supernatant was taken from each microtube into the wells of a plate. 160 $\upmu$l of the Biuret reagent was added and the reaction mixture was incubated for 10 min. The optical density was measured at a wavelength of 540 nm with Thermo Scientific Multiskan FC (Thermo Fisher Scientific Inc., USA).

### Determination of the SPI hydrolysis kinetics on a semi-industrial scale

Hydrolysis of 10% SPI (Shansong 90) was carried out in a homogenizer “Shredder-mixer IS-5” (Limited Company small-scole innovation Enterprise “BioPischeMash”, Russia; The Core Facilities Centre of Federal Research Center “Pushchino Scientific Center for Biological Research of the RAS”) (Budrik and Shchipunov 2021) with constant stirring at a speed of 1100 rpm with dispersing header. The working volume of the homogenizer is 4 liters. When studying the dependence of the hydrolysis rate on the temperature of the reaction mixture, the temperatures 30, 40, 50 and 60 ^∘^C were used, other experiments were performed at 40 ^∘^C. When studying the kinetic dependence of hydrolysis at different pH values, the hydrolysis was carried out in phosphate buffer (pH 3.0 and 5.0) and borate buffer (pH 9.0). The pH of the reaction mixture was measured during the hydrolysis reaction, and it was not corrected in the course of incubation. Sampling was performed after 0, 10, 30, 60, 90 and 120 min. After taking the first sample (zero point), PS was added to the reaction mixture in the amount equal to 6.3 units of activity per 1 g of SPI. When studying the dependence of the hydrolysis rate on the activity of PS, the activity values of 1.6, 6.7, 11.9 and 17.7 units per 1 g of SPI were used. The samples were centrifuged at 12,100 g for 5 min at room temperature. Next, 100 $\upmu$l of the supernatant was taken from each tube and mixed with 400 $\upmu$l of 5% TCA to stop the hydrolysis reaction. The contents of the tubes were mixed and incubated for 10 min, and then centrifuged for 3 min at 12,100*g*. 40 $\upmu$l of the supernatant was taken from each microtubes into the wells of a plate and 160 $\upmu$l of the Biuret reagent was added. The reaction mixture was incubated for 10 min. The optical density was measured at a wavelength of 540 nm with Thermo Scientific Multiskan FC (Thermo Fisher Scientific Inc., USA). The reaction mixture in the homogenizer was heated to 70 ^∘^C for 10 min in the end of incubation to inactivate PS, after that the mixture was cooled to 20 ^∘^C, frozen and freeze-dried for chromatographic analysis.

### Drying the SPI hydrolysate samples

Freeze-drying of the SPI hydrolysate was carried out using a lyophilizer LS-1000K (ProInTech Ltd., Russia). Spray-drying was carried out using a spray dryer BIORUS-8000 (LLC Bio-Rus, Russia) at 130, 150 and 170 ^∘^C with the spray nozzle diameter 0.75 mm and the spraying speed 1 l/h. The obtained samples were compared with size exclusion chromatography as described in the next subsection.

### Size exclusion chromatography

The protein was determined by size exclusion chromatography on the TSK-Gel Filtration Column G3000SW in the Agilent 1100 system with a diode array detection unit (G1315B, Agilent, Waldbronn, Germany) at 220 nm, according to the recommendations of the manufacturer of the chromatographic column. 0.3 M NaCl in 0.05 M phosphate buffer (pH 7.0) with a flow rate of 1 ml/min was used as the mobile phase. 40 ml of 1% protein solution or hydrolysate dissolved in the mobile phase was applied to the column.

Both native SPI and its hydrolysate chromatograms were normalized along the zero line and integrated with the integration boundaries corresponding to the peaks of molecular masses markers.

### Statistical analysis of the results

When determining the kinetics of hydrolysis, the arithmetic mean and standard deviation were calculated for 8 samples for the laboratory scale and 9 samples for the semi-industrial scale. The graphs show mean values and errors equal to two standard deviations. The Tukey method was used to determine outliers and exclude them from the sample (Tukey 1977).

## Results and discussion

In this paper, the dependence of the efficiency of soy protein isolate hydrolysis with the enzyme preparation Protosubtilin G3x (PS) under various conditions of laboratory and semi-industrial procedure (pH, number of units of activity per amount of substrate, temperature of the reaction mixture) was studied.

### pH of the reaction mixture

The acidity level of the reaction mixture can significantly affect the efficiency of protein hydrolysis. In this regard, the pH of the mixture is maintained at a certain optimal value in laboratory protocols and in the technological cycle in industry. In the food industry, the list of used buffer systems is very limited. Therefore, the effect of pH on the hydrolysis efficiency should be checked in each individual case of hydrolysis of a certain protein by a certain proteolytic enzyme.

Figure 2 shows the kinetic dependencies of the accumulation of the protein hydrolysis product in buffer solutions with different pH values, as well as in deionized water, on a laboratory and semi-industrial scale.

On a laboratory scale (Fig. 2a), there are no statistically significant differences between reactions in buffer solutions (pH 5.29, 6.00 and 8.62) up to 60 min of incubation. In deionized water, a sharp increase in the content of TCA-soluble peptides is observed in the interval from 10 to 30 min from the moment of adding the enzyme preparation. After 60 min, the curve of accumulation of peptides reached a plateau. In the reaction mixture without using a buffer system, by the 60 min of incubation, 2 times more reaction product was accumulated than for reactions in buffer solutions.

For the semi-industrial scale, changes in the pH of the reaction mixture during the reaction are also shown (Fig. 2b). Thus, the pH of the reaction mixture after the dissolution of SPI in a buffer solution with a pH of 3.0 was 5.42, and during hydrolysis it decreased to 5.34. For a buffer solution with a pH of 5.0, having received the dissolved SPI in the buffer the value was 6.16, and during the reaction it decreased to 6.01. For a buffer with a pH of 9.0 the pH value decreased to 8.60 after the dissolution of SPI and to 7.86 during the reaction. The maximum rate of accumulation of TCA-soluble peptides is achieved at the pH of the buffer solution of 5.0. However, the difference between all the obtained values is statistically insignificant. An experiment with measuring pH during hydrolysis in a deionized water (without the use of a buffer solution) the pH of the reaction mixture takes the value 8.0 and decreases to 7.2 (Fig. 2c) that is close to the optimal pH for Alcalase 2.4L FG (maximally active at pH 7–10 Lorenz (1978)).

In many previous published works both with PS Zinchenko et al. (2018); Muranova et al. (2018, 2019) and Alcalase Lorenz (1978); Meinlschmidt et al. (2015); Ma et al. (2015) buffer systems are required to maintain the optimal pH for hydrolysis. Also buffer systems are used in the most part of patents (US4100024, US4100024, US7300680B2, US7332192B2, US20060134310A1, US20070269554A9, US20080096243A1). According to our results we claim that deionized water is more preferable than buffer systems in the process of SPI hydrolysis on the semi-industrial scale. We suggest that the high concentration of protein works as a buffer system itself.

### The number of proteolytic activity units in the reaction mixture

The need to determine the dependence of the reaction rate on the number of units of proteolytic activity in the reaction mixture has an economic justification. It is required to use the minimum amount of the enzyme in the reaction mixture to obtain the necessary level of protein hydrolysis within the technological production cycle. In addition, a multicomponent substrate (food protein) may contain specific and non-specific inhibitors of proteolytic enzymes, so the parameter under discussion is selected in each specific case. The mass ratio of substrate to enzyme by mass, often used in the literature, is not correct because of the inability to standardize the process for this parameter due to the high dispersion of the activity value in the volume from batch to batch of the enzyme preparations.

The dependence of the accumulation of product reaction on the number of protosubtilin activity units was determined during 2 h (Fig. 3).

In a laboratory-scale experiment, a significant difference in the yield of the reaction product is observed between all the selected values of the number of units of enzymatic activity in the mixture (Fig. 3a).

In a semi-industrial experiment, after 2 h of incubation, a significant difference is observed only for the lowest activity value (1.6 units per 1 g of SPI). The use of 6.7, 11.9 and 17.7 units per 1 g of SPI leads to the same yield of the reaction product (Fig. 3b). Thus, to conduct the hydrolysis the optimal value of proteolytic activity is 6.7 units per 1 g of SPI for 120 min. A further increase in the activity of PS in the reaction mixture is not required if there is no need to reduce the hydrolysis time. This can be explained by the intensive stirring during the operation of a rotor–stator type mixer used for the semi-industrial scale experiments.

In the previous works with PS the researchers operated with enzyme/substrate ratio (w/v) Zinchenko et al. (2018). We operated with units of PS protease activity so it is not able to compare our results with these articles.

In the part of patents with Alcalase the recommended activity is 2.4 AU/g of SPI (US7300680B2, US20070269554A9) or 4-25 AU/g of SPI (US4100024). In other patents it is not defined (US4324805, US5716801, US7332192B2, US20060134310A1, US20080096243A1, US20080182002A1, US5520935).

**Fig. 4:**
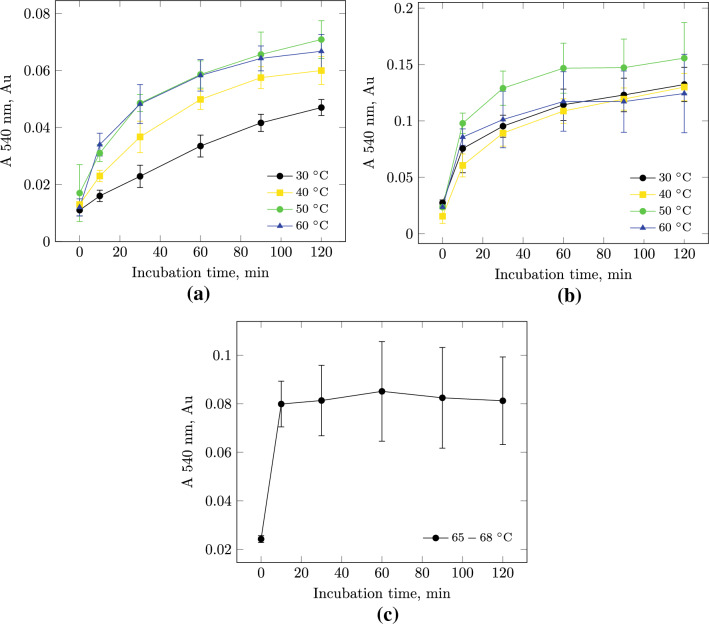
Kinetic dependence of the accumulation of TCA-soluble peptides during the hydrolysis of soy protein isolate under the action of protosubtilin at different temperatures (the reaction mixture contained 6.7 units of proteolytic activity of PS per 1 g of SPI): **a** on a laboratory scale,** b** semi-industrial scale, **c** semi-industrial scale at 65–68 ^∘^C

**Fig. 5:**
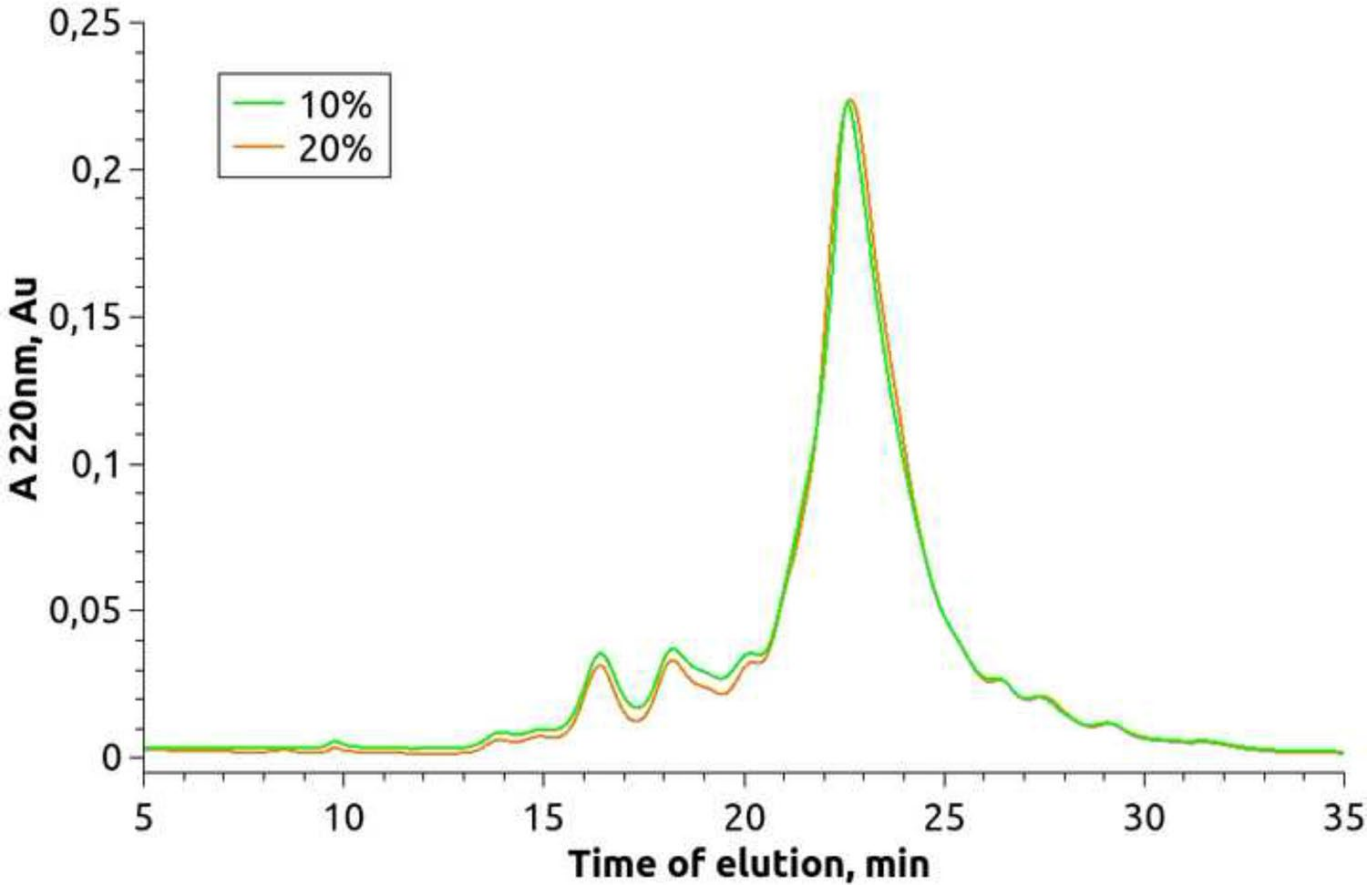
Chromatogram of 10% and 20% SPI hydrolysate. The SPI hydrolysates were obtained by enzymatic hydrolysis of  SPI isolate Shansong 90 using commercial enzyme Protosubtilin G3x with protease activity 11.9 Units/g of SPI at 50 ^∘^C with no buffering

**Fig. 6:**
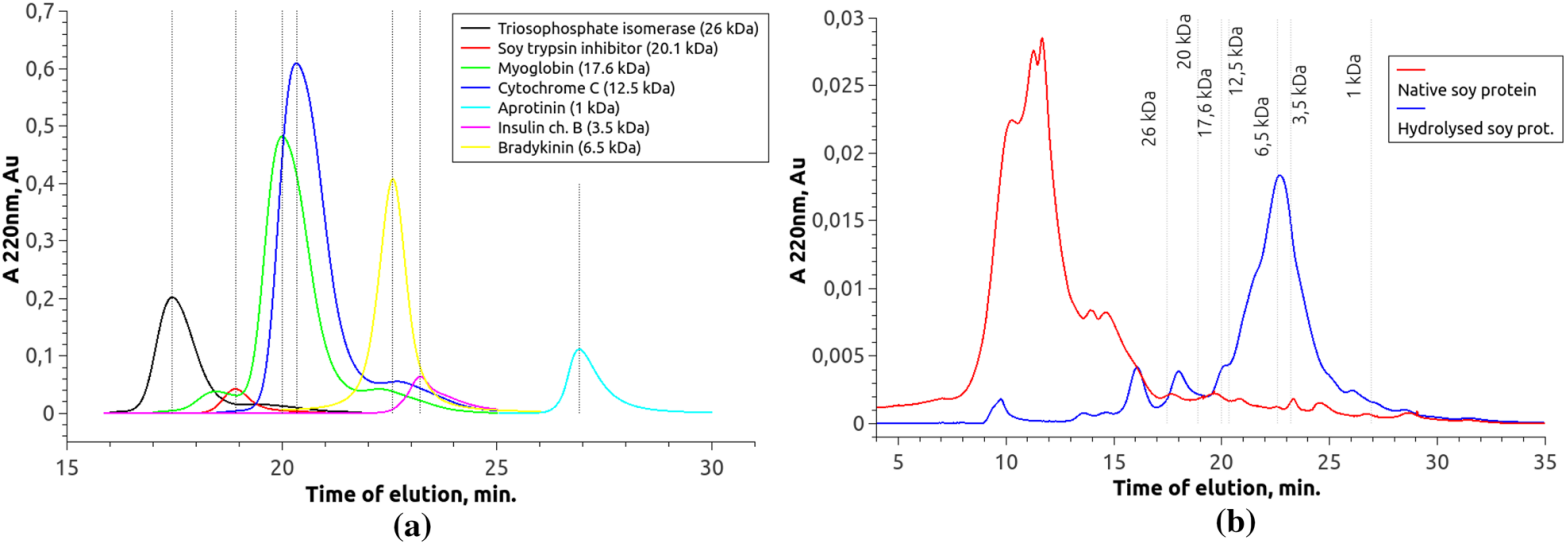
Chromatogram of native SPI, SPI hydrolysate, as well as proteins and peptides with known molecular weights. **a** Chromatogram of proteins and peptides with known molecular weights;** b** comparison of the output time of native SPI components and SPI hydrolysate. The vertical lines correspond to the peaks of the markers on (**a**). The SPI hydrolysate was obtained by enzymatic hydrolysis of 20% SPI isolate Shansong 90 using commercial enzyme Protosubtilin G3x with protease activity 11.9 Units/g of SPI at 50 ^∘^C with no buffering

**Fig. 7:**
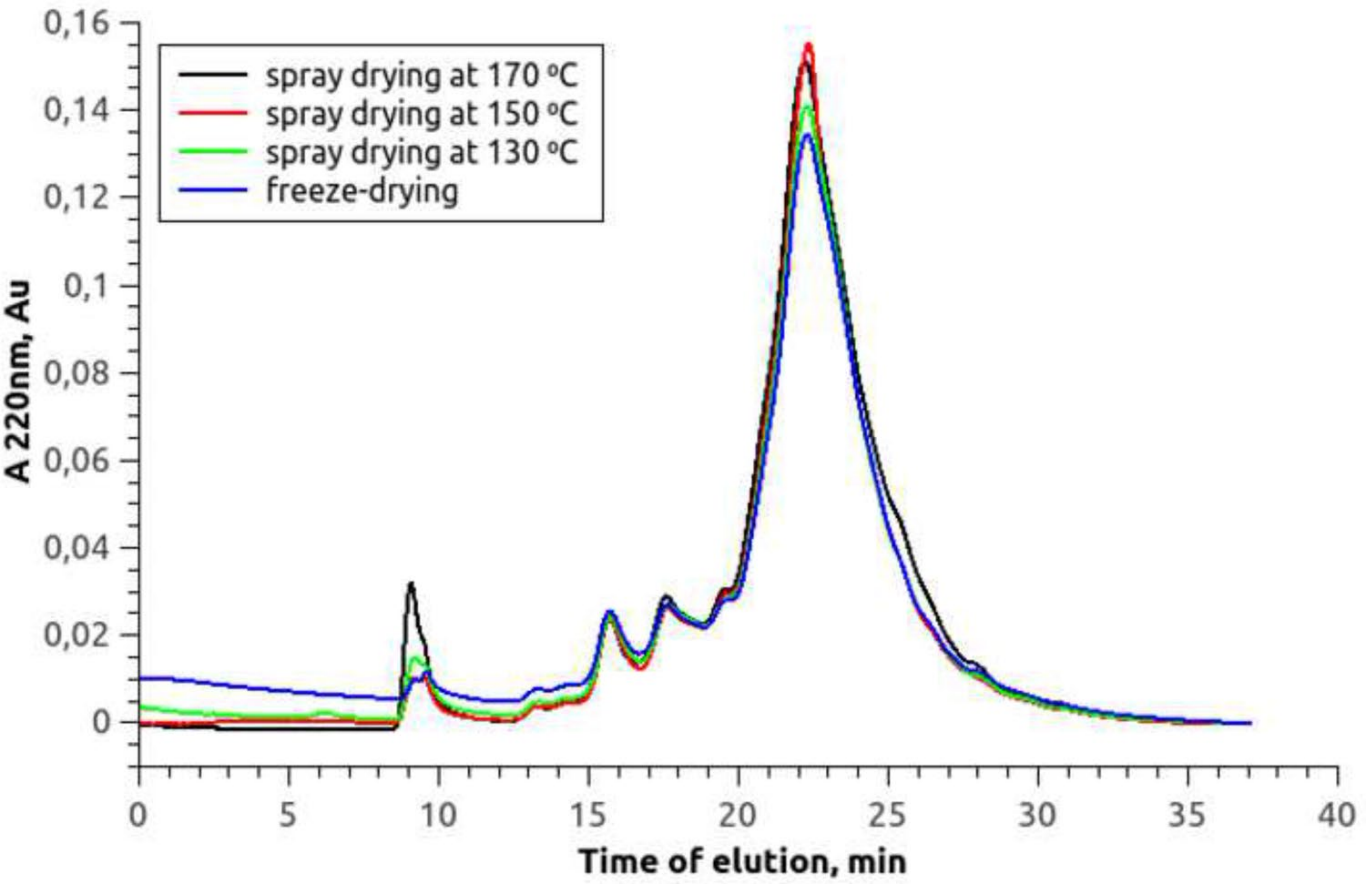
Chromatograms of SPI hydrolysate after freeze and spray drying at different temperatures The SPI hydrolysate was obtained by enzymatic hydrolysis of 20% SPI isolate Shansong 90 using commercial enzyme Protosubtilin G3x with protease activity 11.9 Units/g of SPI at 50 ^∘^C with no buffering

**Fig. 8:**
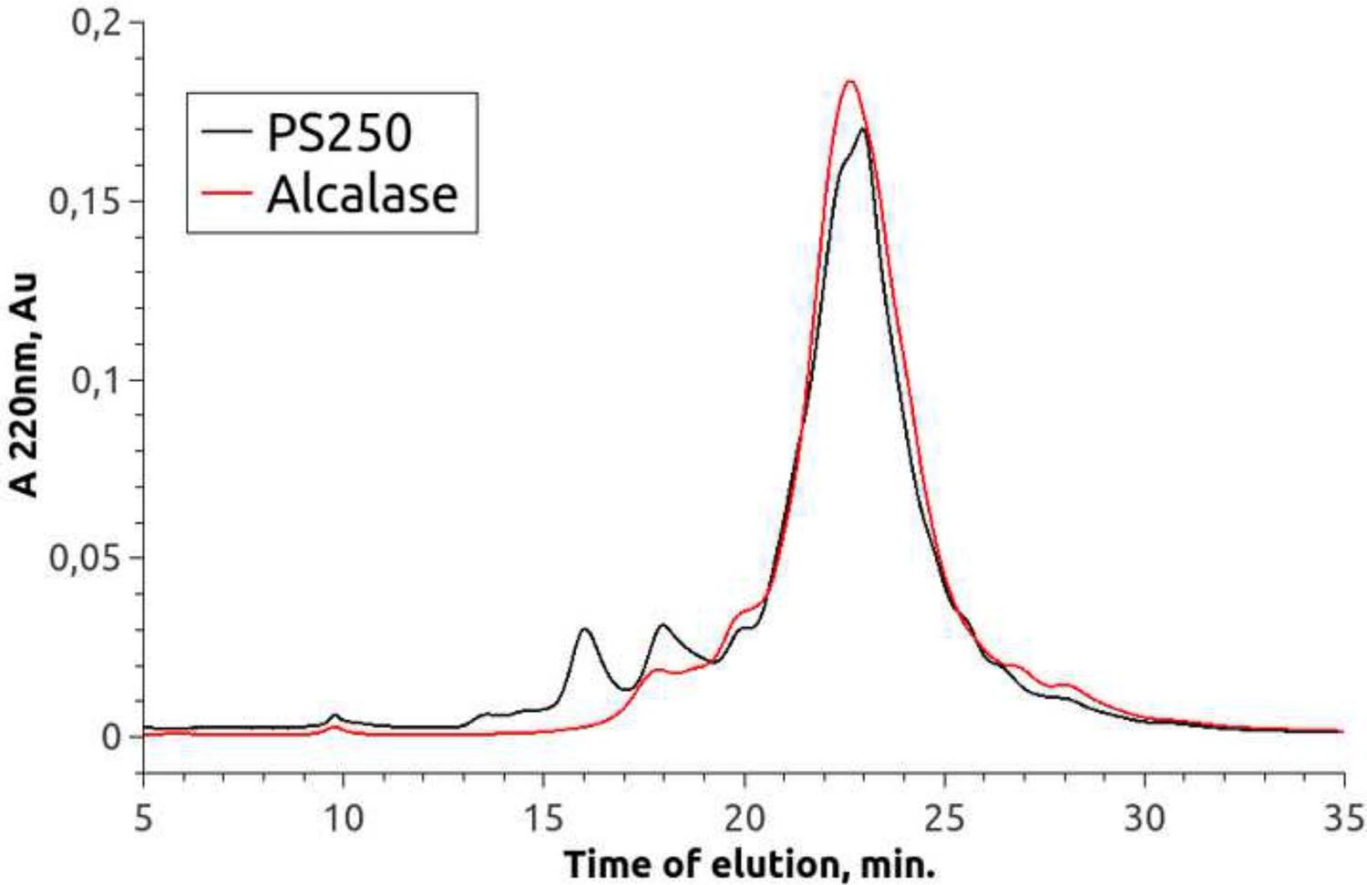
Chromatograms of SPI hydrolysate after treatment with Alcalase and Protosubtilin The SPI hydrolysate was obtained by enzymatic hydrolysis of 20% SPI isolate Shansong 90 using commercial enzymes Alcalase and Protosubtilin G3x with protease activity 11.9 Units/g of SPI at 50 ^∘^C with no buffering

### The temperature of the reaction mixture

The temperature of the reaction mixture affects the activity of the enzyme and the solubility of the substrate, which means that it affects the reaction rate. Just like the pH and activity of the enzyme in the reaction mixture, the temperature is selected for each individual case of enzymatic hydrolysis of the protein. The temperature of deactivation of the enzyme is also determined to stop reaction in the end of incubation. The experiments were carried out at 30, 40, 50 and 60 ^∘^C in deionized water (Fig. 4).

On the laboratory scale of the experiment there was not significant difference in the content of TCA-soluble peptides between samples with a reaction temperature of 40, 50, and 60 ^∘^C (Fig. 4a). The lowest increase in the content of TCA-soluble peptides is observed at 30 ^∘^C.

On a semi-industrial scale, the optimal temperature for carrying out enzymatic hydrolysis of SPI in the presence of PS is 50 ^∘^C. The plateau of the kinetic curve is observed 60 min after the start of the reaction (Fig. 4b). At other temperatures, the kinetic dependence of the SPI hydrolysis reactions on time do not have statistically significant differences from each other. When hydrolyzing SPI at a temperature of 65–68 ^∘^C, the reaction stopped after 10 min. Thus, it was concluded that the enzyme was deactivated (Fig. 4c).

This result matches with researches both for PS Zinchenko et al. (2018) and for Alcalase Lorenz (1978). It was reported that the highest degree of hydrolysis and protein recovery values were obtained at 60 ^∘^C Ma et al. (2015). In patents it is recommended to perform hydrolysis with Alcalase at the ranges of temperatures that include 50–60 ^∘^C (US4100024, US5716801, US7332192B2, US20060134310A1, US20070269554A9, US20080096243A1).

### Size exclusion chromatography of the native soy protein isolate and its hydrolysate

The main characteristic of the hydrolysis by endopeptidases is the size distribution of the resulting peptides. The same characteristic is used as the main one when standardizing the technology for obtaining a hydrolysis product on an industrial scale. Therefore, a comparative analysis of proteins and peptides of native SPI and its hydrolysate obtained on a semi-industrial scale was conducted by size exclusion chromatography. Proteins and peptides with a known molecular weight were used as molecular mass markers (Table 1). For this experiment, a product sample was used, which was obtained by hydrolysis of 20% SPI with an enzyme preparation of PS (11.9 units per 1 g of SPI) at 50 ^∘^C without pH control in the reaction mixture.

**Table 1:** Molecular weights of standard proteins and peptides

Protein or peptide	Molecular weight, kDa
Bradykinin	1
Insulin	3.5
Aprotinin	6.5
Cytochrome C	12.5
Myoglobin	17.6
Soybean trypsin inhibitor	20.1
Triosephosphate isomerase	26

Figure 6a shows a chromatogram of proteins and peptides with known molecular weight. The output time of the components of SPI and its hydrolysate (Fig. 6b) was compared with the output time of the markers. Then the SPI and hydrolysate chromatogram sections located between the peaks of the marker output were integrated. The results are presented in Table 2 as the percentage of proteins and peptides in certain ranges of molecular weights. The percentage was derived from the total mass of the sample, for which the result of integrating the entire chromatogram was taken.

**Table 2:** Distribution of molecular weights of proteins and peptides of native and hydrolyzed soy protein isolate (SPI) according to size exclusion chromatography results

Range of molecular weights, kDa	Native SPI, %	SPI hydrolysate, %
< 1	1.7	5.5
1–3.5	2.8	24.7
3.5–6.5	0.6	15
6.5–12.5	2.4	32
12.5–17.6	0.5	1.9
17.6–20.1	1.7	3.7
20.1–26	2.1	5.7
> 26	88.2	11.6

Table 2 shows that about 88% of the components of native SPI have a molecular weight more than 26 kDa and 11.8%—less than 26 kDa. This result matches the earlier publication that declares that 80–94% of protein fractions in soybean cultivars have a molecular weight more than 12 kDa Oomah et al. (1994).

In the SPI hydrolysate, 88.5% of the components have a molecular weight below 26 kDa. At the same time, 77.1% of the hydrolysate components have molecular weight below 12.5 kDa. The obtained results matches with the earlier publication qualitatively Zinchenko et al. (2018). The suggested way of quantitative analysis could be used for the standardization of the commercial product.

### Effect of SPI concentration on the distribution of molecular weights of peptides of SPI hydrolysate

In the food industry, spray drying is often the final stage of the technological chain for obtaining powdery substances. First of all, this is due to the ease of transportation and storage of the finished product. Spray drying is more effective if the concentration of substrate is high in suspension before drying. Thus, it is necessary to study how the increase of SPI concentration in the reaction mixture will the change product composition.

The distribution of molecular weights of components of two SPI hydrolysates has been compared using size exclusion chromatography. The first hydrolysate has been obtained by enzymatic hydrolysis of 10% SPI isolate Shansong 90 at 40 ^∘^C, the second hydrolysate is a product of enzymatic hydrolysis of 20% SPI isolate Shansong 90 at 50 ^∘^C. In both cases the protease activity of PS was 11.9 Units/g.

Figure 5 shows no significant difference between the products so we can make a conclusion that neither concentration of SPI in the reaction mixture nor temperature changes the distribution of molecular weights of components of the two SPI hydrolysates. In the next studies the hydrolysate obtained by the enzymatic hydrolysis of 20% SPI with PS (11.9 units/g of SPI) at 50 ^∘^C with no bufferization has been used.

### Effect of spray drying temperature conditions on the distribution of molecular weights of peptides of SPI hydrolysate

The spray drying is based on treating the product with high temperature in a chamber, where the product enters as a fine aerosol. This method of removing water is economically justified in comparison with the use of the sublimation method. However, treatment of the SPI hydrolysate with high temperature can change its peptide size profile due to thermal hydrolysis. Therefore, using the size exclusion chromatography method, the sizes of hydrolysate peptides were compared after freeze and spray drying at different processing temperatures.

Figure 7 shows that the chromatograms of all samples practically do not differ from each other. Thus, the size profile of the peptides of the SPI hydrolysate does not depend on the drying method. In addition, the results demonstrate the reproducibility of the obtained size exclusion chromatography data (see chromatograms of samples after freeze-drying in Figs. 6 and  7).

### Comparison of Protosubtilin and Alcalase

Alcalase 2.4L FG is a commercial enzyme obtained from *Bacillus licheniformis*, permitted for the food industry. The protocol of the SPI hydrolysis with Alcalase was developed by Adler-Niessen Lorenz (1978) (US4100024), and the later study showed that it is the most suitable commercial enzyme for this purpose Meinlschmidt et al. (2015).

In this study we use Protosubtilin G3x (PS) that is obtained from the culture fluid of *Bacillus subtilis* and used for fodder only. The optimal parameters recommended by the manufacturer differs for these two enzymes, but in our study we show that PS works better in the conditions recommended for Alcalase. Also, it is known, that the main component of both commercial products is subtilisin.

Figure 8 shows no significant difference between the products of SPI hydrolysis with Alcalase and PS.

We suggest that PS could become an analogue of Alcalase but several actions are required. PS appears to be carrier powder with the culture fluid of *Bacillus subtilis* sprayed on it, that’s why it is impossible to use it for the production of peptides directly so filtration and purification steps are nessessary. Exclusion of the carrier powder or its replacement to a nutritional substance could significantly facilitate the application of PS in the food industry.

## Conclusions

In this work, optimal conditions for the hydrolysis of soy protein isolate with the enzyme preparation Protosubtilin G3x (PS) were found. The difference in the obtained results on a laboratory and semi-industrial scale is shown. Thus, the optimal temperature range was established. On a laboratory scale it is 40–60 ^∘^C. On a semi-industrial scale the optimal value is 50 ^∘^C. In addition, the optimal number of enzyme activity units was established on a laboratory scale, namely 11.8 units per 1 g of SPI. When scaling, it was shown that it is possible to reduce this value to 6.7 units per 1 g of SPI without losing the hydrolysis efficiency.

In the future, we are going to study the bioavailability of the obtained SPI hydrolysate on animal model. We propose that the occurrence of free amino acids in blood after feeding and physical activity could be a marker of the bioavailability of the product.

In most studies devoted to the study of protein hydrolysis, including those for soy protein, researchers pay a lot of attention to maintaining a certain pH value during the reaction. In our study, there was no statistically significant dependence of the hydrolysis reaction on pH. Thus, it can be concluded that in the case of SPI hydrolysis by an enzyme preparation of PS, maintaining the pH in the reaction mixture is not required. This can save time and reagents needed to prepare buffer solutions on an industrial scale, as well as reduce the risk of industrial injuries when working with large volumes of reagents needed to maintain the pH of the reaction mixture.

The type of equipment used in this work for semi-industrial scale hydrolysis is scaled up to hundreds of liters of working volume. Thus, it can be suggested that the obtained results of semi-industrial experiments can form the basis of industrial regulations for the production of enzymatic hydrolysate of soy protein isolate. The observed differences in the results of laboratory and semi-industrial experiments emphasize the necessity of semi-industrial experiments to accelerate the transfer of scientific achievements to the industrial sector of the economy.

## Data Availability

All data generated or analysed during this study are included in this published article.

## Abbreviations

FAO: Food and Agriculture Organization of the United Nations
PS: Protosubtilin G3x
SPI: soy protein isolate Shansong 90
TCA: trichloroacetic acid
WHO: World Health Organization
